# Strategies for Using the Sheep Ovarian Cortex as a Model in Reproductive Medicine

**DOI:** 10.1371/journal.pone.0091073

**Published:** 2014-03-10

**Authors:** Maïté Fransolet, Soraya Labied, Laurie Henry, Marie-Caroline Masereel, Eric Rozet, Nathalie Kirschvink, Michelle Nisolle, Carine Munaut

**Affiliations:** 1 Laboratory of Tumor and Developmental Biology, GIGA-R, University of Liège, Tour de Pathologie (B23), Sart Tilman, Liège, Belgium; 2 Analytical Chemistry Laboratory, CIRM, Institute of Pharmacy, University of Liège, and Arlenda s.a., Sart Tilman, Liège, Belgium; 3 Veterinary Integrated Research Unit, Faculty of Sciences, Namur Research Institute for Life Sciences (NARILIS), University of Namur (UNamur), Namur, Belgium; 4 Department of Obstetrics and Gynecology, University of Liège, Hôpital la Citadelle, Liège, Belgium; Faculty of Animal Sciences and Food Engineering, University of São Paulo, Pirassununga, SP, Brazil, Brazil

## Abstract

**Objective:**

To evaluate and compare the distribution and density of primordial follicles within a whole sheep ovary and to gain insight into how to overcome the impact of natural follicular heterogeneity on the experimental results.

**Design:**

Histological study.

**Setting:**

Academic research center.

**Animals:**

Five- to nine-month-old ewes.

**Interventions:**

Freshly sampled whole sheep ovaries were collected and prepared for histological analysis.

**Main Outcome Measure(s):**

The follicular densities and distributions were determined for hematoxylin and eosin sections. A mathematical model was derived based on the follicle counts and Monte-Carlo simulations.

**Results:**

Heterogeneous distributions and densities of primordial follicles were identified 1) for distinct areas of the same ovarian cortex, 2) between the ovaries of the same animal and 3) across different ewes. A mathematical model based on the analysis of 37,153 primordial follicles from 8 different ovaries facilitated the estimation of the number of cortical biopsies and sections that had to be analyzed to overcome such heterogeneity.

**Conclusion:**

The influence of physiological follicular heterogeneity on experimental and clinical results can be overcome when a definite number of cortical pieces and sections are taken into consideration.

## Introduction

Ovarian cryopreservation is a promising technique for fertility preservation in young women with cancer prior to sterilization by chemotherapy or radiotherapy. This technique creates the hope of restoring gonadal function in iatrogenically sterilized patients upon autografting [1], [2]. Moreover, ovarian cryopreservation is the most appropriate strategy when embryonic cryopreservation is not feasible [3].

Fifteen years of clinical advances in ovarian tissue cryopreservation and cryobanking demonstrate that the procedure is safe, easy and promising. The pregnancy rate after the autografting of cryopreserved tissue to orthotopic sites is estimated to be approximately 30%, although the precise denominator is currently unknown [4]. To date, approximately 24 infants have been born worldwide using this procedure [5].

Even if human ovarian tissue has been occasionally used in studies of animal models [6], ethical barriers and the limited availability of human ovarian tissue preclude the widespread use of human ovaries for research. Consequently, there is an unmet need for a robust animal model comparable with humans. The “ideal” animal model should be similar to humans in terms of biochemical, physiological and anatomical characteristics. The greatest contribution of animal models, especially those of nonhuman primates, is that they offer the ability to conduct controlled experiments that would be logistically or ethically proscribed in women [7], [8]. In the case of cryopreservation studies, additional criteria for suitability as an animal model research tool would be similar ovarian architectures, a limited number of developed follicles and a single ovulation with primordial follicles distributed superficially in the cortex [9]. The sheep ovary is an adequate candidate for research according to these criteria. Its size is 80% of the human premenopausal ovary [9], [10], [11], and it has a comparable collagen-dense cortical layer containing the pool of primordial follicles. Indeed, the sheep ovary has been used for many studies [12], [13], [14], [15], [16].

The pool of human primordial follicles in cryopreserved ovarian cortical biopsies is of major importance for the subsequent restoration of fertility. The cryopreservation of live human ovarian tissue requires that cortical slices be no more than 1 mm thick. When the follicular reserve is low, this technical limitation decreases the likelihood of adequate follicle collection and increases the risk of subsequently transplanting empty biopsies. In women, follicle density and follicle distribution have been described as being highly variable according to the age and physiologic status [17], [18], [19], [20]. Different strategies have been pursued to facilitate the assessment of follicle density *in situ* as a research tool to ensure that only tissue pieces with abundant follicles are used [21], [22]. No precise data, methodology or guidelines for ovarian tissue analysis are available.

The purpose of the present study was to evaluate and compare the distribution and density of primordial follicles within a whole sheep ovary. Furthermore, we established a mathematical model to estimate the number of cortical pieces or sections that should be analyzed to limit the impact of follicular heterogeneity on experimental results.

## Materials and Methods

### 1) Collection and preparation of ovarian tissue

The Animal Ethics Committee of the University of Namur approved the use of sheep ovarian tissue. Eight ovaries harvested from four ewes (5 ½, 7, 6 and 9 months old) were obtained from the Ovine Research Center (University of Namur). After euthanasia, the ovaries were immersed in Leibovitz L-15 medium (Lonza, Verviers, Belgium, BE12-700F) supplemented with 10% normal sheep serum and transported at 4°C to the laboratory within 1 h. For each ovary, the medulla was removed, and the cortex was cut into small pieces (2.5×2.5×1 mm). The cortical fragments were fixed in 4% paraformaldehyde, embedded in paraffin and fully cut into 5-µm-thick serial sections stained with hematoxylin and eosin (H&E) (Tribune Stainer HCS 33).

### 2) Virtual images acquisition

Virtual images were acquired with the fully automated digital microscopy system dotSlide (Olympus, BX51TF, Aartselaar, Belgium) coupled with a Peltier-cooled high-resolution digital color camera (1376×1032 pixels) (Olympus, XC10, Aartselaar, Belgium). Digital images of the whole tissue sections were digitized at high magnification (100×), producing virtual images with a pixel size of 1.510 µm.

### 3) Ovarian cortex analysis

The whole scanned H&E sections were analyzed using Image J software. The follicles were quantified manually, and to avoid double counting, only follicles with visible nuclei were taken into account. Follicles were then classified according to their maturity (primordial, primary or secondary follicles), as previously described [23]. The follicular densities (number/mm^2^) were calculated after manually outlining the cortical surface (Image J software).

### 4) Statistical analysis

To model the impact of the ewes, ovaries and ovarian fragments on the primordial follicle density, a linear mixed model was applied to the density of primordial follicles 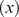 after subjecting the variable to the following base-10 logarithmic transformation: 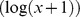. All data analyses were performed using JMP v10.0 (SAS institute, Cary, USA).

## Results

### 1) Primordial follicle distributions and densities within whole sheep ovaries

**Figure 1:**
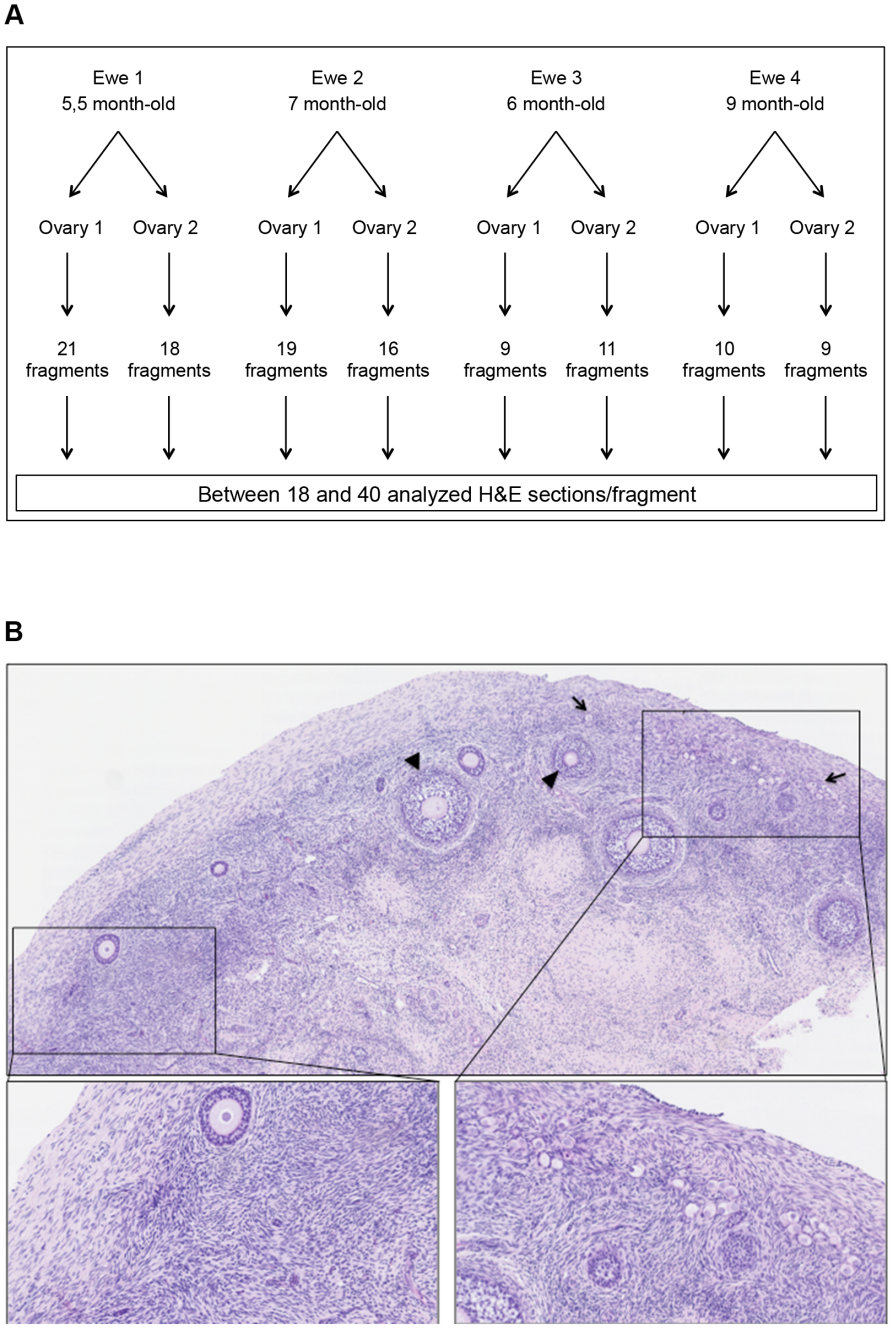
Flow chart of the experimental design (A) and representative histology of a sheep cortex section (B). Ovaries were harvested from two ewes and fully cut into cortical fragments. Subsequently, each fragment was serially and completely sectioned, and approximately 40 H&E sections, each 30 µm apart from one another, were further used for the follicular quantification (A). The uneven repartition of follicles within the sheep ovarian cortex is obvious (B). The left part of the H&E section is completely devoid of primordial follicles, whereas the right part contains mostly primordial follicles. Primordial follicles (plain arrows) and secondary follicles (arrowhead).

To assess the primordial follicle distributions in the sheep cortex, we analyzed the whole ovaries using the procedure depicted in the flow chart presented in Figure 1A. Both ovaries from 4 young ewes were collected. After removal of the medulla, the remaining cortex was cut into small fragments (9–21 fragments/ovary). Each fragment was completely serially sectioned (5 µm thick). Because sheep primordial follicle diameters range from 20 to 30 µm, we analyzed every sixth section of the serially sectioned ovarian cortex fragments. In total, 3,852 H&E sections were analyzed. Figure 1B illustrates one H&E section of a hemi-ovary where uneven primordial follicle distribution is obvious.

**Figure 2:**
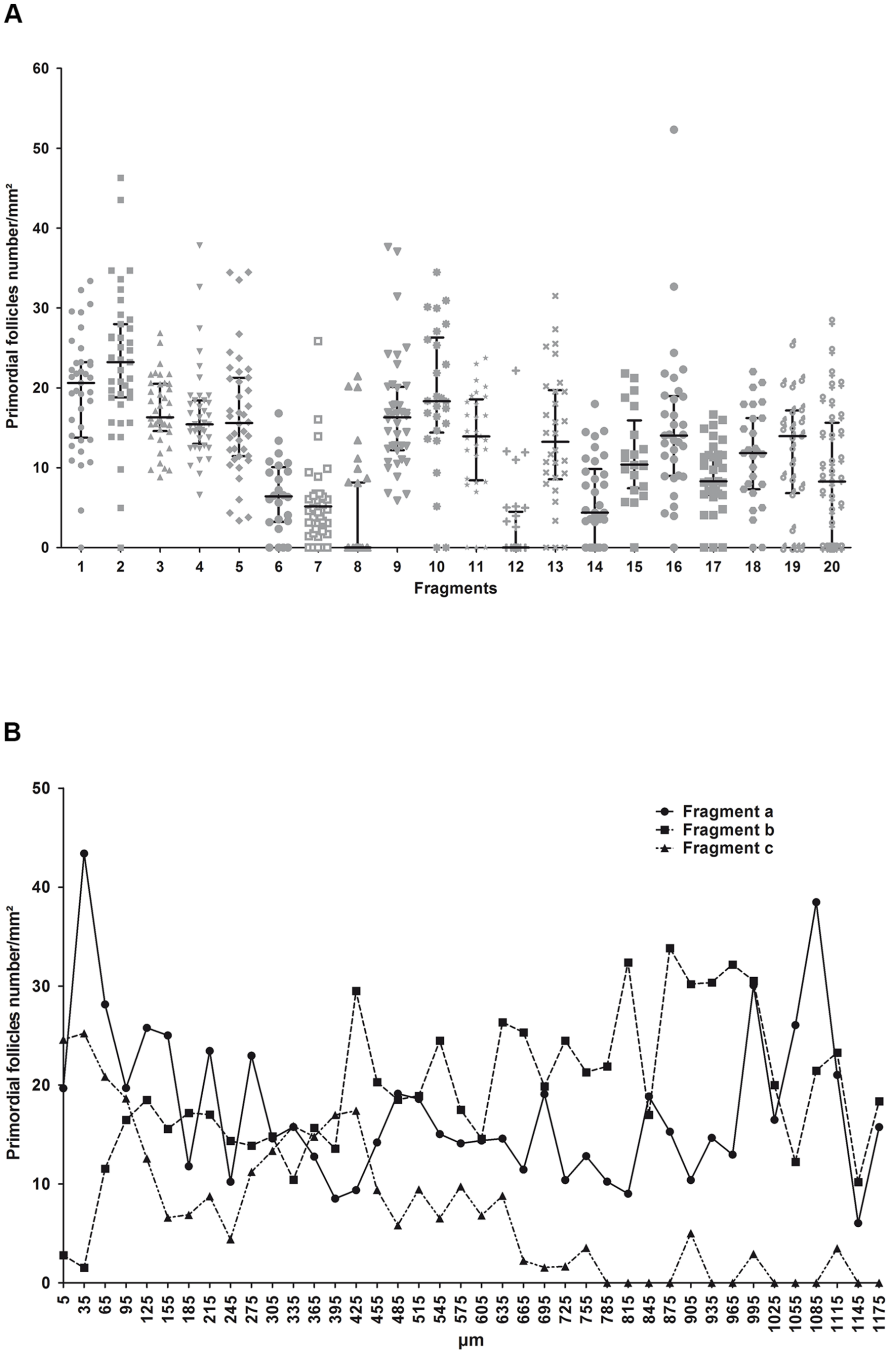
Representative illustration of the primordial follicular quantification. Mean primordial follicle densities (number/mm^2^) of 40 sections per fragment for 20 fragments from the same ovary (A) are shown. An example of the primordial follicle density within serial sections (at a 30-µm distance) of three fragments from the same ovary is also illustrated (a, b and c) (B).

In this study, we only quantified primordial and primary follicles (Table 1). Secondary follicles representing less than 1% of the entire population were not taken into account. Figure 2 illustrates the primordial follicle density analysis for a single whole sheep ovary. The primordial follicle density is highly variable both between fragments belonging to the same ovary (Figure 2A) and among serial sections belonging to the same fragment (Figure 2B). In each ovary, there was a wide variation in the number of primordial follicles across the fragments. This analysis highlights the importance of adequately determining the number of fragments or sections necessary to overcome physiological follicular heterogeneity.

**Table 1:** Follicle quantification in the whole ovaries.

	H&E sections analyzed	Primordial follicle number	Primary follicle number
Ewe 1, ovary 1	657	5624	325
Ewe 1, ovary 2	669	8572	317
Ewe 2, ovary 1	632	7746	235
Ewe 2, ovary 2	567	6808	628
Ewe 3, ovary 1	301	2409	619
Ewe 3, ovary 2	360	2369	320
Ewe 4, ovary 1	378	2345	267
Ewe 4, ovary 2	288	1285	267
**Total**	**3,852**	**37,153**	**2,978**

### 2) Mathematical model

To create a linear mixed model of the follicle counts, the ewes and ovaries were treated as fixed factors. The ovaries were nested into the ewes, and the ovarian fragments were treated as random factors. After logarithmically transforming the primordial follicle densities, the weights of the fixed factors could be determined: the ewes had a significant effect on the primordial follicle density (p-value<0.0001). This finding indicated that the primordial follicle density was not identical between the two ewes. Moreover, the ovaries had a significant effect on the primordial follicle density (p-value<0.0001). Indeed, the follicular density was different between the two ovaries collected from the same animal. These findings indicated that in an experimental setup aiming to study the effects of specific treatments on follicular density, a single ovary should be used to limit the impact of the fixed factors (such as ewes and ovaries). Alternatively, if several ewes or several ovaries are required, then their effects should be included in the statistical model to extract their interferential influences over the treatment effects.

In a specific ovary, 9 to 21 pieces of cortex were analyzed, and the histological evaluation of the primordial follicle density revealed a wide variation among the sections of the same fragment (Figure 2). When ovarian fragments were treated as random factors with the residual error representing the number of sections per ovarian fragment, the statistical analysis clearly showed that the major source of variability was due to the sections per ovary fragment, accounting for 90% of the total variance (Table 2). The residual variability linked to the fragments accounted for only 10% of the total variance. This finding suggests that to increase the precision of the primordial follicular density measurement, increasing the number of sections per fragment rather than the number of analyzed fragments is more rewarding.

**Table 2:** Variance components of the random-effects fragments and sections and their respective standard errors.

Random effects	Variance component	Standard error	Proportion of variance
Fragment	0.116	0.037	10.46±3.00%
Section	0.993	0.023	89.54±2.06%

In the ovarian cortex, the primordial follicles represented the most important follicle population; therefore, we based the rest of our statistical analysis on their density. The primary follicles, however, displayed the same distribution profiles and density heterogeneities (data not shown).

### 3) Determination of the sample size

Based on the results obtained from the analysis of the follicular densities, Monte-Carlo simulations were performed to determine the best experimental design required to evaluate the effects of a specific ovarian treatment. The same linear mixed model as described for the analyses above was used. The logarithmically transformed primordial follicle densities were hence supposed to follow a normal distribution. Estimations of the effect of the ewes and ovaries obtained from the previous statistical analysis were used as fixed values in the model to define the mean of the distribution used for simulation. The estimated variance components of the ovarian fragments and sections were used to define the variance of the logarithmically transformed primordial follicle densities. An additional fixed factor was added to the mean of the model corresponding to a treatment effect that could have an impact on the follicle densities. The treatment fixed effects (i.e., the effects of a treatment on follicular density that should be detected) were arbitrarily set at 50, 25 and 10% modification of follicular densities with respect to a control treatment. For each of these three possibilities, 2000 simulations were performed for different combinations of numbers of ovarian fragments (20, 10 or 5) and numbers of sections per ovarian fragments (40, 20 or 10) defining the sample size. The probability of detecting the prespecified effect size on follicular density, i.e., the power of the test, was computed (Table 3). In the simulations, the treatment effect was defined as being significant for p-values<0.05. If a 50% improvement in the follicular preservation is required, all experimental setups that were tested allowed for detecting this improvement with 100% probability. To detect a more subtle effect, e.g., 25% follicular preservation with at least 95% probability, two experimental setups out of five allowed us to limit the total number of analyzed sections to 200 per group: either 10 fragments and 20 sections per fragment or 20 fragments and 10 sections per fragment. To power the system to detect an improvement in follicular preservation as small as 10%, at least 20 fragments with 40 sections per fragment had to be examined for the probability of detecting the effect to remain above 80%.

**Table 3:** Results of the Monte-Carlo simulations.

Fixed effect (%)	Number of fragments	Number of sections	Probability of detecting the effect (%)
50	20 or 10 or 5	40 or 20 or 10	100
25	20	40	100
25	20	20	100
***25***	***20***	***10***	***99***
25	10	40	100
***25***	***10***	***20***	***98***
25	10	10	93
25	5	40	94
25	5	20	87
25	5	10	74
10	20	40	84
10	20	20	71
10	20	10	54
10	10	40	70
10	10	20	53
10	10	10	39
10	5	40	56
10	5	20	44
10	5	10	29

The probability of detecting an effect on the primordial follicular density (i.e., power) was calculated with the fixed effects arbitrarily set at 50, 25 and 10% and a confidence level of alpha  = 0.05 for various combinations of the numbers of ovary fragments and sections per fragment.

## Discussion

We used a methodologically rigorous and laborious research technique to measure follicular distribution within the ovine ovarian cortex. Serial sections of different fragments covering several whole sheep ovaries displayed uneven primordial follicle densities, as has already been reported for the human cortex [17], [19], [20]. In our study, 3,852 sections belonging to 113 cortical fragments extracted from eight ovaries were analyzed and subsequently used to build a mathematical model useful for further application in the field of reproductive medicine. The primary focus of this study was not to perform comparisons among individuals; our specific aim was to provide guidelines regarding the number of samples and histological sections required to achieve specific objectives in the improvement of follicular cryopreservation.

Currently, the cryopreservation of cortical fragments followed by transplantation represents the only opportunity for fertility preservation available to young women with cancer who need immediate chemotherapy. However, this strategy is still considered an experimental technique that requires improvement. The initial follicle density within cryopreserved ovarian tissue has a profound impact on the transplantation success. The lifespan of the graft depends on the number of preserved viable primordial follicles [24], [25].

The study of follicle preservation is still hampered by the lack of a clearly defined experimental model that takes into account the physiological heterogeneity within an ovarian cortex. A diagnostic test that has the ability to confirm the presence of follicles and to indicate the survival of cryopreserved follicles would be a valuable tool in the field of reproductive medicine, particularly for ovarian tissue cryopreservation. The accuracy of estimating the follicle density in the human ovary based on a single biopsy or sample has been questioned [26], [27], [28], and studies have shown large variations in follicle density between and within ovaries when multiple samples are examined [19] or a lack of homogeneous follicle distribution even within a single sample [17], [29], [30], [31]. *In situ* follicle identification methods should be non-detrimental to the tissue and should allow long-term follicle survival and growth. Several methods of follicle detection using stereomicroscopic localization, fluorescent probes (Calcein AM or rhodamine 123) and the dye neutral red (NR) have been established [21], [22], [32]. All of these methods for follicle visualization have their own advantages and disadvantages (e.g., the expense and specificity of the required equipment, operator experience, toxicity toward oocytes and the thickness of cortical pieces [less than 500 µm]). Therefore, none of these methods is suitable for evaluating the follicular density in frozen-thawed fragments prior to transplantation or for analyzing transplants after a defined period of transplantation.

Our histological analysis followed by mathematical simulation provides useful information for establishing a follicular quantification method that considers the physiological follicular heterogeneity existing within the ovarian cortex and between ovaries or individuals (Tables 2 and 3). This method can be usefully applied in the field of ovarian tissue transplantation as an option for fertility preservation. For example, to perform *in vivo* or *in vitro* treatment comparisons, a sufficient number of cortical pieces from the same ovary of the same animal with a sufficient number of serial sections or fragments should be analyzed.

Additionally, our study clearly indicates that the number of ovarian fragments and/or the number of sections/fragments should be increased proportionately to the decreased expected effect (Table 3). This method is also useful for determining the number of host animals to be grafted for obtaining results that are independent of the follicular heterogeneity.

In conclusion, our study offers a valuable tool for evaluating the efficacy and safety of multiple treatments that may be beneficial for follicular preservation within xenograft models, using either human or sheep ovarian tissue.
